# Systemic application of teriparatide for steroid induced osteonecrosis in a rat model

**DOI:** 10.1186/s12891-015-0589-z

**Published:** 2015-07-11

**Authors:** Yulei Dong, Yulong Li, Cheng Huang, Kai Gao, Xisheng Weng

**Affiliations:** Deparment of Orthopaedic Surgery, Peking Union Medical College Hospital, Chinese Academy of Medical Science and Peking Union Medical College, 100730 Beijing, China; Institute of laboratory animal sciences, Chinese Academy of Medical Science and Peking Union Medical College, 100021 Beijing, China

**Keywords:** Steroid induced osteonecrosis, Teriparatide, Parathyroid hormone

## Abstract

**Background:**

Steroid associated osteonecrosis is difficult to treat. Teriparatide, as the only one bone anabolic drug, has achieved very promising effect in osteoporosis and other bone skeletal diseases. We carried out this animal study to evaluate the effect of subcutaneous injection of teriparatide for the steroid induced femoral head necrosis in a rat model.

**Methods:**

24 Sprague–Dawley male adult rats were included in the study. All the rats were randomized into 4 groups: 18 rats from LPS/MPS group, LPS/MPS + PTH group and LPS/MPS + NS group were given lipopolysaccharide (20 μg/kg) and methylprednisolone (40 mg/kg) to establish the steroid induced osteonecrosis model. 6 rats from NS group only received normal saline. 4 weeks later, All the rats in LPS/MPS group and NS group were sacrificed and the femoral heads were harvested. After that, the 6 rats in the LPS/MPS + PTH group received subcutaneous injection of 20 μg/kg teriparatide and LPS/MPS + NS group only received equal amount of normal saline. After 4 weeks, the serum bone marker was tested and the femoral head were harvested. Micro-CT and histological examination were performed to compare the incidence of osteonecrosis and trabeculae parameters for the femoral head.

**Results:**

At 4 weeks, rats in LPS/MPS group showed significant osteonecrosis by histological examination (83.3 %) which suggested successful steroid induced osteronecrosis animal models were established. After the treatment of 4 weeks, the LPS/MPS + PTH group showed significant lower incidence rate of osteonecrosis compared with the LPS/MPS + NS group (16.7 % vs.75 %, P < 0.05). The micro CT examination showed higher bone volume/total volume, trabecula thickness and bone mineral density in the LPS/MPS + PTH group compared with the LPS/MPS + NS group. The serum osteocalcin was a little higher in the LPS/MPS + PTH group (4.54 ± 1.61vs.3.58 ± 1.81, *P* = 0.358), but it didn’t reach a statistical significance.

**Conclusions:**

Systemic application of teriparatide for steroid induced osteonecrosis in rats showed a beneficial effect. This may be one promising therapy for early stage osteonecrosis.

## Background

Osteonecrosis of the femoral head was a side effect of the corticosteroid in treating autoimmune and other diseases. High-dose corticosteroid administration was considered to be the most common risk factor for osteonecrosis [1]. The progression of osteonecrosis would ultimately lead to collapse of the femoral head when femoral head or total hip replacement was usually required. Osteonecrosis was responsible for 10 % of all total hip replacement surgeries and was the most common cause of total hip replacement in young adults; but problems with infection, osteolysis, dislocation and revisions were worse with total hip replacement for steroid-induced osteonecrosis than for osteoarthritis [2]. Lots of conservative therapy including anticoagulants, bisphosphonates and statins had been tried on the early stage femoral head necrosis [3–5], but there was still no satisfactory resolution.

Teriparatide (rhPTH[1–34]) was a bone anabolic agent which was approved to treat osteoporosis both in women and men [6]. In vitro studies had shown that teriparatide stimulated new bone formation by increasing osteoblast number, enhancing osteogenic differentiation of bone mesenchymal stem cell and improving cell survival [7]. Besides osteoporosis, teriparatide had emerged as a potential curative drug in promoting spinal fusion [8], bone fracture healing [9] and biphosphates associatedosteonecrosis of the jaw [10]. One of the pathogenesis of femoral head necrosis was that the steroid suppressed the activity of the bone mesenchymal stem cell and decreased the osteogenic differentiation [11]. So we hypothesized the teriparatide might treat the steroid induced femoral head necrosis and carried out the prospective experiment in a rat model.

## Methods

### Animals

The experiment was performed under the approval of the Animal Experimentation Ethics Committee of Peking Union Medical College Hospital. Twenty-four Sprague–Dawley male adult rats (age for 12 weeks; body weight for 400–450 g) were used in this study. The SD rats were bought from Charles River Company (Beijing, China). All rats were housed in pairs in custom-designed plexiglas cages (50 × 35 × 20 cm) under standard laboratory conditions (12/12-h light/dark cycle, 24-25 °C, humidity with 50-55 %) and allowed freely access to food and water during the study.

### Grouping and animal model establishment

**Fig. 1:**
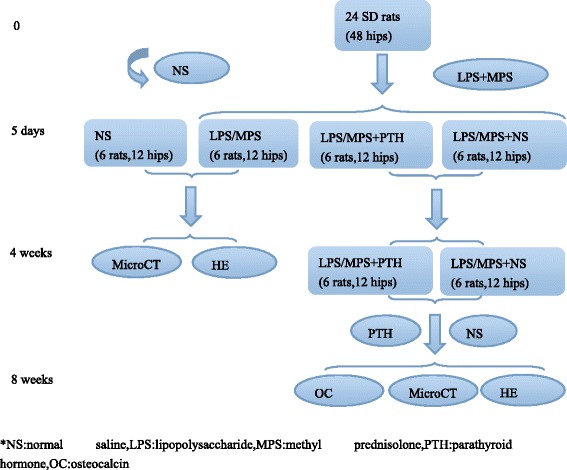
The flow diagram of the study

All the rats were randomized into 4 groups: NS group, LPS/MPS group, LPS/MPS + PTH group, LPS/MPS + NS group.(see Fig. 1).18 rats from the latter three groups were given two doses intraperitoneal injection of 20 μg/kg of lipopolysaccharide (LPS, Escherichia coli 055:B5, Sigma, St. Louis, MO, USA) on days 0 and 1 at a time interval of 24 h, 24 h later, the eighteen rats received three intramuscular injection of 40 mg/kg methylprednisolone Sodium Succinate (Pfizer Pharmaceutical, China) on days 3, 4 and 5 at a time interval of 24 h. 6 rats from NS group only received normal saline. 4 weeks later, All the rats in LPS/MPS group and NS group were sacrificed and the femoral heads were harvested. The femurs were decalcified and stained with HE. The successful osteonecrosis animal model was confirmed by histological examination.

The twelve rest rats in the LPS/MPS + PTH group and the LPS/MPS + NS group would continue to be kept under the same condition. The former group received 20 μg/kg of teriparatide (Lilly, Indianapolis, USA) subcutaneous injection everyday, while the latter group received normal saline. After four weeks, the blood samples were collected from the inferior vena cava with the animals in a fasting state. Then, the rats were sacrificed with an overdose of pentobarbital sodium (240 mg/kg ip) and bilateral femurs were harvested.

### Serum osteocalcin examination

The serum of the rat blood was collected by centrifugation at 3000 rpm for 10 min at 4 °C and stored at −80 °C until examination. Serum osteocalcin was measured by ELISA using a kit from SenXiong Technologies Incorporated (Shanghai, China). This protocol adopted the method of double antibody sandwich ABC-ELISA. With anti-rat Osteocalcin monoclonal antibody coated on the ELISA plate, anti-rat Osteocalcin biotinylated antibody was added, and immune complexes would connected to the board. Streptavidin labeled with horseradish peroxidase would bind to biotin, then enzyme substrate TMB was added, it would turn blue, then stopping solution of sulfuric acid was added, the color would turn yellow. Finally the OD value was measured at 450 nm. As the rat Osteocalcin concentrations was proportional to the OD value, the osteocalcin concentration in rat specimens could be obtained by drawing standard curve. Each specimen would be repeated for three times.

### Micro CT scan and quantitative analysis

**Fig. 2:**
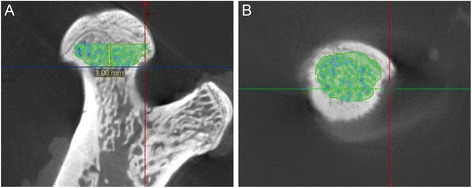
The region of interest chosen and the identification of trabecula

The femur specimens were sampled and fixed in 10 % buffered neutral formalin solution until examination. The specimens were scanned by Inveon micro PET/CT manufactured by Siemens (Berlin, Germany) at a voltage of 60 kV and a current of 400uA, with entire scan length of 20 mm in a spatial resolution of 10 μm used for animal experimental studies and reconstructed using Inveon analysis workstation. The micro CT diagnosis standard for femoral head necrosis is fracture of the trabecula, cyst degeneration, sclerosis band or flattened shape of the femoral head. Two independent researchers made the diagnosis blindedly, if there was disagreement, consensus was reached by a third person. 1 mm below the center of the epiphyseal line with a total of 50 slices was chosen as the region of interest (ROI) for the analysis and comparison of trabecula paramenters. On the cross-section the cortical bone and spongy bone was separated manually by auto trace, later the trabeculae and the bone marrow was separated by the threshold function (Fig. 2). After that, the bone volume/total volume(BV/TV), bone surface area/bone volume, trabecular thickness, trabecular number, Trabecular spacing, trabecular pattern factor, cortical wall thickness and bone mineral density (BMD) in the ROI were computed respectively by the workstation.

### Histopathology

The femur specimens were fixed in 10 % buffered neutral formalin solution for 72 h and followed by decalcification with 10 % ethylenediaminetetraacetic acid (EDTA)- 0.1 M phosphate buffer (pH 7.4). After decalcification, the tissues were dehydrated in graded ethanol, embedded in paraffin, cut into 4 μm thick sections in the coronal plane, processed for routine hematoxylin and stained by eosin for the evaluation of osteonecrosis. All sections were assessed blindly by two independent authors using a microscope (Leica, Wetzlar, Germany), the diagnosis of osteonecrosis was established based on the presence of empty lacunae or pyknotic nuclei of osteocytes in the bone trabeculae, accompanied by surrounding bone marrow cell necrosis [12]. If the diagnosis differed between the two examiners, a consensus was reached by a third person.

#### Statistical analysis

The Student’s *t* test or Fisher’s Exact test was performed to compare the variables between the two groups. The parameters were expressed as Mean ± SD. Statistical analysis was performed using SPSS for Windows (Version 17.0; SPSS Inc., Chicago, IL). A P <0.05 was considered as significant difference.

## Results

### Histological examination

**Fig. 3:**
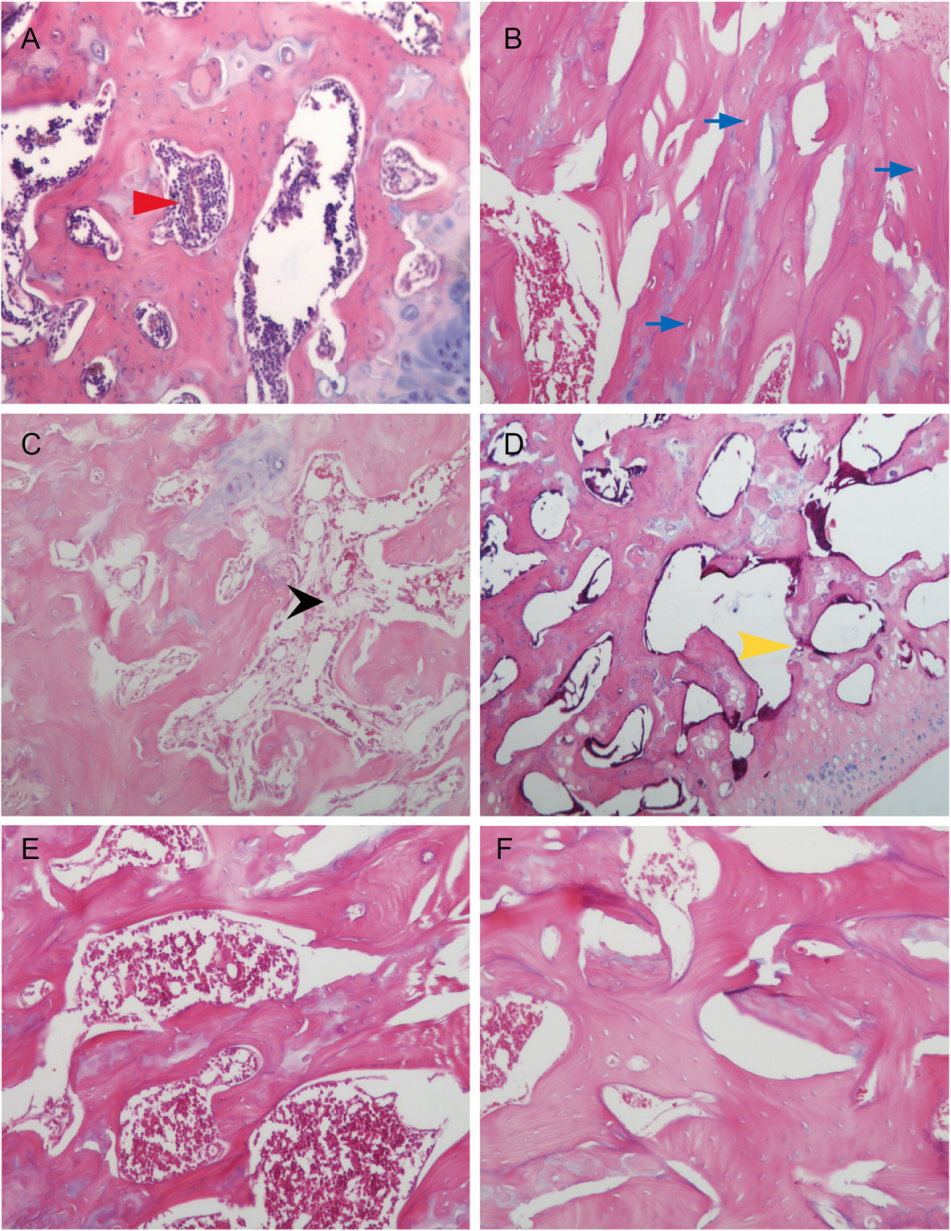
The histopathological appearance of the femoral head. **a** was from NS group which showed well arranged trabecula and normal osteocyte with nuclei. The red arrow demonstrates the normal bone marrow. **b**, **c** and **d** were from LPS/MPS group which showed empty bone lacuna, bone marrow necrosis, and broken trabecula. The blue arrow in **b** demonstrateds the empty bone lacuna. The black arrow in **c** demonstrates the necrosis of the bone marrow. The yellow arrow in **d** demonstrates the broken of the trabecula. At 8 weeks, E from LPS/MPS + NS group showed broken of the trabeculae. **f** was from LPS/MPS + PTH group which showed better arranged and more condense trabeculae compared with E.(HE stained; magnification,×100)

All rats were survived during the experimental period. The histopathological appearance of the femoral head was given in Fig. 3. The empty bone lacuna and broken of the trabeculae were obvious in the LPS/MPS group. The incidence of osteonerosis at 4 weeks was 83.3 %(10/12) in the LPS/MPS group and 0(0/12) in the NS group by the standard of HE stained histological examination.(P < 0.05) which suggested a successful animal model of steroid induced femoral head necrosis was established. By the HE stained histological examination, the incidence of osteonecrosis was 75 %(9/12) in the LPS/MPS + NS group and 16.7 %(2/12) in the LPS/MPS + PTH group at 8 weeks. (P < 0.05) The incidence of osteonerosis of LPS/MPS + PTH group was significantly lower than the LPS/MPS + NS group.(see Table 1). Morover, the histopathological examination showed that the trabeculae in the LPS/MPS + PTH group was condense and well arranged while the trabeculae in the LPS/MPS + NS group was thin and disorderly.

**Table 1:** Incidence of osteonecrosis(%)

Group	Week 4 HE	Week4 CT	Week 8 HE	Week8 CT
LPS/MPS group	83.3(10/12)	58.3(7/12)	NA	NA
NS group	0(0/12)	0(0/12)	NA	NA
LPS/MPS + PTH group	NA	NA	16.7(2/12)	0(0/12)
LPS/MPS + NS group	NA	NA	75(9/12)	50(6/12)
P value	0.000	0.005	0.012	0.014

### Serum osteocalcin

The serum osteocalcin showed a little higher in the LPS/MPS + PTH group than the LPS/MPS + NS group, however, the result didn’t reach a statistical significance (Table 2).

**Table 2:** The comparison of serum osteocalcin between the two groups

	LPS/MPS + NS group(n = 6)	LPS/MPS + PTH group(n = 6)	T value	P
1	4.921	4.365		
2	6.634	2.092		
3	2.487	5.741		
4	2.927	6.802		
5	2.562	4.195		
6	1.953	4.017		
mean ± SD	3.58 ± 1.81	4.54 ± 1.61	−0.96	0.358

### Micro CT scan and quantitative analysis

**Fig. 4:**
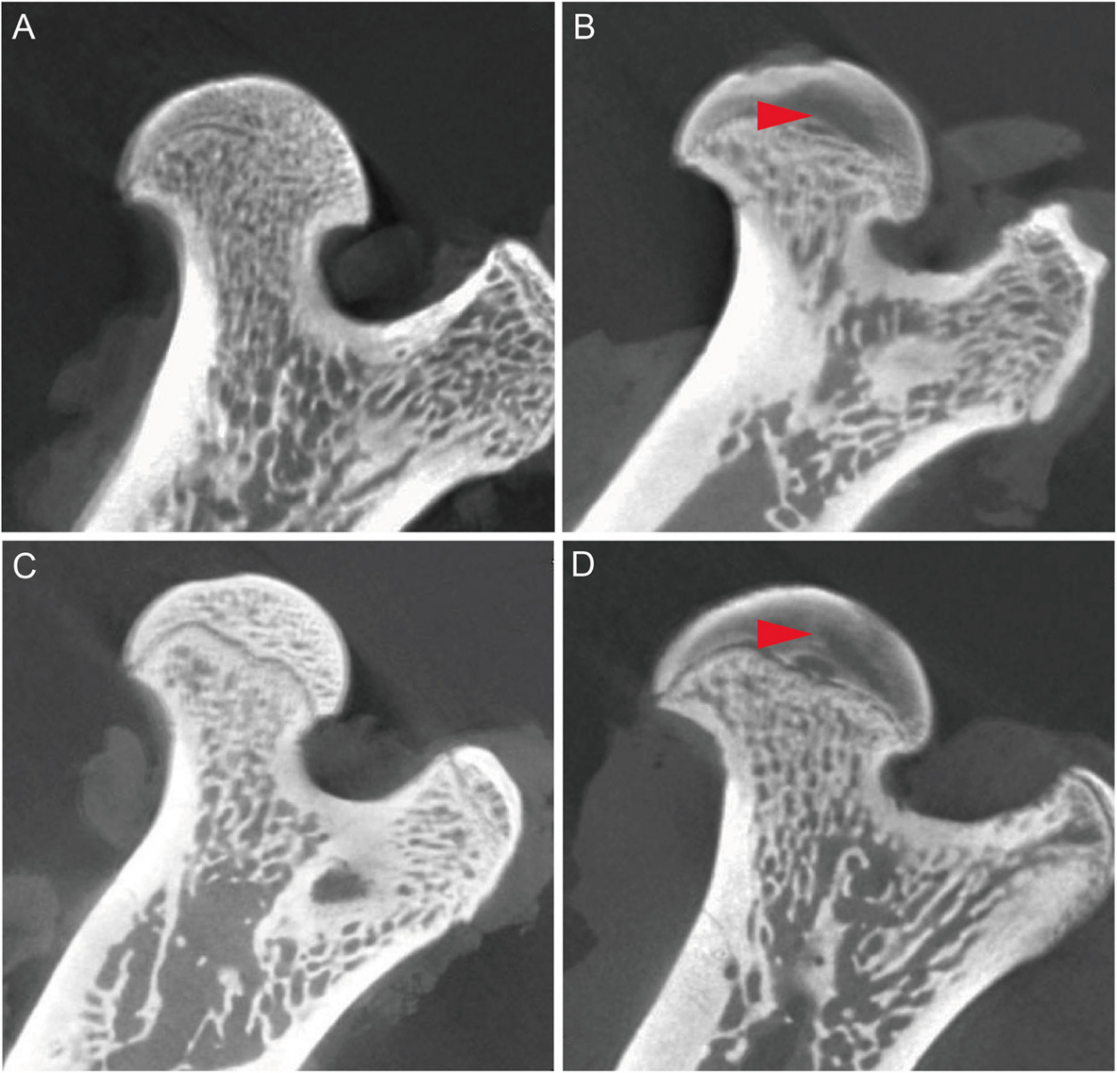
The coronal section of the femoral head. **a** was from NS group. **b** was from LPS/MPS group. **c** was from LPS/MPS + PTH D was from LPS/MPS + NS group. Obvious necrosis and cyst degeneration could be seen in the **b** and **d**(red arrow). In the LPS/MPS + PTH, no significant osteonecrosis lesion was seen

By the standard of microCT, the incidence of osteonecrosis was 58.3 %(7/12) and 0(0/12) in the LPS/MPS group and NS group respectively (P < 0.05). At 8 weeks, the incidence of osteonecrosis was 50 %(6/12) in the LPS/MPS + NS group and 0(0/12) in the LPS/MPS + PTH group(P < 0.05). The incidence of osteonerosis of LPS/MPS + PTH group was significantly lower than the LPS/MPS + NS group.(see Table 1). Representative micro CT images of osteonerosis were given in Fig. 4.

The quantitative analysis of the trabeculae showed that bone volume/total volume, trabecula thickness and bone mineral density were significantly higher in the LPS/MPS + PTH group (Table 3).

**Table 3:** The comparison of trabecula parameters between the two groups

	LPS/MPS + NS group(n = 12)	LPS/MPS + PTH group(n = 12)	T value	P
Bone Volume/Total Volume	0.55 ± 0.11	0.65 ± 0.11	−2.26	0.035
Bone Surface Area/Bone Volume	22.42 ± 5.20	17.30 ± 4.78	2.46	0.023
Trabecular Thickness	0.09 ± 0.03	0.12 ± 0.03	−2.27	0.034
Trabecular Number	5.87 ± 0.48	5.39 ± 0.77	1.76	0.094
Trabecular Spacing	0.08 ± 0.02	0.07 ± 0.02	1.68	0.107
Trabecular Pattern Factor	−0.47 ± 4.59	−4.20 ± 3.38	2.23	0.037
Cortical Wall Thickness	0.60 ± 0.12	0.54 ± 0.12	1.29	0.212
Bone Mineral Density(mg/cc)	350.84 ± 79.93	447.49 ± 53.72	−3.431	0.003

## Discussion

Steroid induced femoral head necrosis was refractory and most of the patients would undergo collapse of the femoral head and had to receive total hip arthoplasty. Many researchers have tried biphosphates, anticoagulant and statins [3–5]. However, there is still no ideal resulotion for the early staged osteonecrosis. Some researchers have described abnormalities in the number or in the function of bone progenitor cells in osteonecrosis [11, 13]. The hypothesis that osteonecrosis could be related to a mechanism that results in an imbalance between osteoblast formation and necrosis developed.[14, 15] Moreover, the osteogenic differentiation of the mesenchymal stem cell was also attenuated by glucocorticoids [16, 17].

In the present study, the steroid-induced osteonecrosis model was established in rats by two injections of low-dose LPS(20ug/Kg) combined with three subsequent injections of high-dose MPS(40 mg/Kg) . The most classical animal model for osteonerosis was rabbit and there were two classical inductive protocols commonly used for establishing ON relevant to steroid. One was to use a single injection of high-dose methylprednisolone (MPS) (20 mg/kg), however, the incidence was as low as 43 %[18]. The other was to use two injections of high-dose lipopolysaccharide (LPS,100ug/Kg) combined with subsequent three injections of high-dose MPS (20 mg/Kg), which induced higher ON incidence (85 %) but accompanied with high mortality of experimental animals (50 %) [19]. Qin et al. applied one intravenous injection of LPS (10 μg/kg) and three injections of 20 mg/kg of MPS. He reported 93 % of the rabbits (13/14) developed ON and no rabbits died throughout the experiment period [20]. Rats were the second common animal osteonecrosis model. Okazaki et al. gave male Wistar rats two injections of 2 mg/kg LPS and three injections of 20 mg/kg methylprednisolone. The incidence of osteonerosis were 67 % at three weeks and 33 % at four weeks. However, the number of the animal was too limited [21]. Tong P et al. applied a dose of 20ug/kg LPS via daily i.p. administration for two times and three injections of methylprednisolone (40 mg/kg). They reported osteonecrosis was observed in seven of ten rats (70 %) [22]. We adopted the method described by Tong P et al. and the incidence of location-specific femoral head necrosis was 83.3 %(10/12) by histological examination. With the standard of microCT, the incidence of femoral head necrosis was 58.3 %(7/12), which was lower than the histological examination. The reason may be that the necrosis of the osteoblast and bone marrow couldn’t be displayed in micro CT images. The histopathology of osteonecrosis in this model was characterized by empty lacunae or pyknotic nuclei of osteocytes accompanied by surrounding bone marrow cell necrosis and the micro CT images of femoral head necrosis were fracture of the trabeculae, cyst degeneration, sclerosis band or flattened shape of the femoral head, which were similar to osteonecrosis in steroid-treated patients. However, osteonecrosis often lead to femoral head collapse in human but not rats, as the epiphyseal line of the femur was permanent in adult rats but not in adult human. What’s more, false negative results may exist with micro CT diagnosis as the early staged osteonecrosis can’t be displayed. This could explain why the incidence of osteoncrosis by CT was lower than that of histology. In our study, the incidence of osteonecrosis in the LPS/MPS group was 83.3 %(10/12), and no rats died during the whole period, indicating that this rat model was safe and reliable. Therefore, this animal model was reliable for evaluating the intervention of steroid-induced osteonerosis studies.

Teriparatide (rhPTH [1–34]) was an amino-terminal fragment of PTH which had been shown to stimulate new bone formation by increasing osteoblast activity and numbers by inducing the mesenchymal stem cell osteogenic differentiation [23]. It was the only anabolic agent currently approved for the treatment of post-menopausal osteoporosis, idiopathic or hypogonadal osteoporosis in men, and steroid-induced osteoporosis [24, 25]. Teriparatide induced new bone formation at inactive bone surfaces and further stimulated mineral apposition at active remodeling surfaces, resulting in increased bone mass trabecular thickness, and improved trabecular connectivity in animals and patients. Histologic studies had shown that the increase in bone formation was largely due to an increase in the number of matrix-synthesizing osteoblasts [26]. Increased osteoblastogenesis, attenuation of osteoblast apoptosis and activation of quiescent lining cells had been proposed as explanations for this effect of PTH.

A great number of studies had shown that the teriparatide had the potential effect in bone union, spinal fusion and bone defect repair [27, 28]. Moreover, teriparatide also had been shown to be useful in periodontal disease. In a trial where 40 adults with chronic periodontitis were subjected to periodontal surgery and teriparatide, it resulted in greater radiographic resolution of the periodontal bone defects compared to placebo with accelerated osseous wound healing in the oral cavity [29]. Osteonecrosis of jaw (ONJ) involved necrotic, exposing bone in the jaw, pain and swelling associated with long-term bisphosphonate use. The precise mechanism underlying the development of ONJ with bisphosphonate use was still not clear. Teriparatide has been reported to be an potential treatment option for ONJ [10]. As teriparatide promotes osteoblast differentiation and activity, it might be beneficial to promote bone formation and necrosis lesion repair.

The pathogenesis of the femoral head necrosis was that the activity of MSC as well as the osteogenic differentiation potential was attenuated by the steroids, application of teriparatide might increase the MSC activity and osteogenic differentiation, thus reverse the effect of steroids in osteonecrosis repair process. Moreover, the trabeculae parameters by micro CT showed that teraparatide also increased the bone volume, trabeculae thickness and bone mineral density of the femoral head, which would reduce the possibility of the femoral head collapse. In the present study, we applied the teriparatide dosage for 20 μg/kg because most of the rodent models reported the use of 5–200 (typically 40) μg/kg BW/day for anabolic effects on skeletal repair. However, the clinical dose for osteoporosis treatment was 20 μg per day for an adult person. Animal models used supraphysiologic doses than that in human. This difference was considered to be derived from species difference. However, it was possible that more than osteoporosis dose might be necessary to treat osteonecrosis in human. Moreover, as for the osteonecrosis, the most suitable course of teriparatide treatment is unclear. The therapy duration in our study was four weeks, and we referred it to the bone union animal model. Alkhiary et al. applied the administration of 30 μg/kg/day of teriparatide in rats with femoral closed fracture showed an improvement in fracture healing since the third week [30]. Nakazawa et al. gave daily subcutaneous injections of 10 μg/kg of recombinant human PTH(1–34) [rhPTH(1–34)] over a 28-day period of fracture healing. On day 14 after fracture, cartilage area in the PTH-treated group was significantly increased [31]. In the spine fusion animal model, Ni Ming et al. applied teriparatide 4 μg/kg per day and teriparatide 23 μg/kg per day subcutaneous injections for 4 weeks (5 days per wk) after the spine fusion surgery in the rat model. They found teriparatide at 23 μg/kg per day for 4 weeks showed anabolic skeletal effects and significantly enhanced spinal fusion rate in rats [32]. However, some researchers also conduct the study with a follow up of 6–8 weeks. Longer course of treatment needed to be studied in further research.

There were still some limits of our study: Firstly, in order to save animals and for animal ethics, we designed the study strictly with the expense of fewer animals. What’s more, as the cost of the teriparatide was expensive, the therapy duration was limited to 4 weeks. However, the location specific analysis of osteonecrosis made up for the limitation as double femoral heads were both evaluated in each rat. Secondly, for human beings, the MRI has emerged as the golden standard of the diagnosis of femoral head necrosis. However, the most common diagnosis standard for rats is histological examination. There are advantages of micro CT with high resolution in evaluating the trabeculae both qualitatively and quantitively, however, the early staged osteonecrosis cann’t be displayed. Finally, the underlying mechanism of teriparatide in osteonecrosis is still unclear, which need to be clarified in further research.

## Conclusions

In general, we present with a prospective randomized animal study which may be the first study exploring the potential effect of teriparatide in the treatment of steroid induced osteonecrosis. Both the micro-CT analysis and the histology results are promising, which is quite illuminating for justifying human studies.

## Abbreviations

SD: Sprague–Dawley
MPS: Methylprednisolone
rhPTH[1–34]: Recombinant human parathyroid hormone[1–34]
LPS: Lipopolysaccharide
ROI: Region of interest
EDTA: Ethylenediaminetetraacetic acid
BMD: Bone mineral density
ONJ: Osteonecrosis of jaw
ELISA: Enzyme-linked immunosorbent assay
TMB: Tetramethylbenzidine
